# The Application of Deep Brain Stimulation for Treatment-Resistant Depression – A Narrative Review

**DOI:** 10.1192/j.eurpsy.2023.2165

**Published:** 2023-07-19

**Authors:** J. Wellington, B. R. Jethnani, Y. A. Elebessy, Y. A. Elebessy, S. M. Abdulrahman, S. Jain, A. M. Jahid

**Affiliations:** 1 Cardiff University School of Medicine, Cardiff, UK, United Kingdom; 2 Newcastle University Medicine, Johor, Malaysia

## Abstract

**Introduction:**

Depression continues to be a leading cause of disability worldwide. Despite the availability of several classes of antidepressants, a third of patients do not recover from their depression. Deep brain stimulation (DBS) is an invasive treatment approach that was found to be effective in the treatment of Parkinson’s Disease and presents as an alternative to standard antidepressant therapy for people with treatment-resistent depression (TRD).

**Objectives:**

We aimed to compare the use of DBS to standard antidepressant therapy and decipher whether DBS can be used for TRD. In addition, electroconvulsive therapy (ECT), a current brain stimulation method administered for TRD, was contrasted with DBS.

**Methods:**

A narrative review of the current literature concerning DBS application and TRD was conducted to evaluate whether standard antidepressant therapy was as effective as psychosurgical intervention. Emphasis on TRD-associated DBS was noted.

**Results:**

The studies discussed found that DBS was an effective treatment option for TRD, however, the results were limited due to the studies being conducted in small sample sizes and using DBS in combination with antidepressant therapy. Nonetheless, the concomitant use of DBS and antidepressants demonstrated to be an effective treatment for TRD, highlighting the potential benefit of DBS in inducing remission in TRD. DBS has a wider range of complications compared to ECT as it involves a more invasive neurosurgical approach to implant the device. On comparing the cost of the devices between the 2 studies, DBS costs approximately three times more than ECT.

**Conclusions:**

The spectrum of depressive disorders is known to affect multiple regions of the brain. A more cohesive approach would be a comprehensive study using DBS in multiple brain regions while incorporating blinded controls. In summary, DBS could be a viable treatment addition for TRD, but more thorough studies are needed to deduce its true efficacy. Future collaborative studies investigating the efficacy of DBS over ECT in TRD may assess further therapeutic potential.

**Disclosure of Interest:**

None Declared

